# Nonendodontic periapical lesions: a retrospective descriptive study in a Brazilian population

**DOI:** 10.4317/medoral.24379

**Published:** 2021-03-27

**Authors:** Giuliana Gismonti Guimarães, Danyel Elias da Cruz Perez, Juliana de Noronha Santos Netto, Ana Camily Oliveira da Costa, Augusto César Leal da Silva Leonel, Jurema Freire Lisboa de Castro, Fábio Ramoa Pires

**Affiliations:** 1 DDS, Post-graduation Program in Dentistry, Estácio de Sá University; 2 DDS, PhD, Oral Pathology, School of Dentistry, Federal University of Pernambuco; 3 DDS, MSc, Stomatology, Estácio de Sá University; 4 DDS, Oral Pathology, School of Dentistry, Federal University of Pernambuco; 5 DDS, PhD, Post-graduation Program in Dentistry, Estácio de Sá University

## Abstract

**Background:**

Several nonendodontic diseases can occur in the periapical region, resembling endodontic inflammatory conditions. Therefore, the aim of the present study was to determine the frequency of nonendodontic periapical lesions diagnosed in a Brazilian population.

**Material and Methods:**

The files of two Oral Pathology laboratories were reviewed and all cases including at least one clinical diagnosis of endodontic periapical lesions were selected for the study. After initial selection, demographic and clinical data, clinical diagnosis and final diagnosis were reviewed and tabulated. Final diagnosis included endodontic periapical lesions, and benign and malignant nonendodontic periapical lesions. Data were descriptively and comparatively analyzed among the three groups, with a significance level of 5% (*p*<0.05).

**Results:**

Nonendodontic periapical lesions were identified in 208 (19%) out of the 1.125 registries included in the final sample. Benign nonendodontic periapical lesions (200 cases, 18%) were mostly odontogenic keratocysts, ameloblastomas, nasopalatine cysts, dentigerous cysts, glandular odontogenic cysts, and benign fibroosseous lesions. Malignant nonendodontic periapical lesions (8 cases, 1%) included carcinomas, adenocarcinomas, and melanoma. In general, nonendodontic periapical lesions were more common in males and in the posterior mandible (*p*>0.05).

**Conclusions:**

The frequency of nonendodontic periapical lesions was high and, although the general distribution was similar to the results from other populations, some features were probably associated with the profile of the studied populations and to the methods applied in the present study. Knowledge on differential diagnosis of endodontic and nonendodontic periapical lesions is essential to avoid unnecessary treatments and diagnostic delay in routine dental practice.

** Key words:**Differential diagnosis, nonendodontic, periapical lesion, pulp necrosis.

## Introduction

Most lesions located in the periapical region are derived from pulp inflammation/necrosis and are collectively called endodontic periapical lesions (EPL) (1). However, several other conditions can occur in the periapical region without association with the pulp status of the adjacent teeth (2). These diseases can resemble both clinically and radiographically the EPL, frequently carrying diagnostic difficulties (2-6).

The group of nonendodontic periapical lesions (NPL) include odontogenic cysts and tumors, fibro-osseous lesions, nonodontogenic intraosseous benign cysts and tumors, and intraosseous malignancies (3,5). These conditions receive specific treatment protocols and, for this reason, it is essential for clinicians and endodontists to consider NPL in the differential diagnosis of periapical diseases. This would avoid the risk of misdiagnosis and inadequate management and/or treatment delay.

Most information on the frequency and clinicopathological aspects of the NPL are derived from case reports and few retrospective studies with large case series. So, the aim of the present study was to access the frequency and distribution of NPL diagnosed in a Brazilian population.

## Material and Methods

This was a descriptive restrospective study. The files of the Oral Pathology Laboratory, School of Dentistry, Rio de Janeiro State University, Rio de Janeiro, Brazil (from 2005 to 2018) and from the Oral Pathology Laboratory, School of Dentistry, Federal University of Pernambuco, Recife, Brazil (from 2000 to 2017) were reviewed. All cases that included a clinical diagnosis of EPL (periapical granuloma, periapical cyst and periapical abscess) were selected and the laboratory registries from each case were reviewed. Information about age and gender of the patients, location of the lesion (maxilla or mandible; posterior or anterior region), symptoms (present or not), local swelling (present or not), clinical diagnosis (the first clinical diagnosis provided by the solicitant), and final diagnosis, were retrieved from the registries and tabulated.

Clinical diagnosis and final diagnosis were individually registered and, for comparative analysis, were grouped in group 1 (all EPL), group 2 (benign NPL - benign odontogenic and nonodontogenic cysts and tumors, developmental defects and nonendodontic infections), and group 3 (malignant NPL).

Data were descriptively and comparatively analyzed among the three groups using the Statistical Program for Social Sciences (SPSS, version 20, IBM) with a significance level of 5% (*p*<0,05) through the use of ANOVA and chi-square.

## Results

A total of 18.167 cases were diagnosed in the two laboratories in the study periods, and 1.125 (6.2%) included an EPL as a clinical diagnosis, composing the final sample. Females (n=590) and males (n=535) represented, respectively, 52% and 48% of the sample. Mean age of the patients was 43 years (SD 17.711), ranging from 4 to 90 years.

Lesions (n=1038) were located in the posterior mandible (301 cases, 29%), anterior maxilla (286 cases, 28%), posterior maxilla (118 cases, 11%), anterior mandible (70 cases, 7%), maxilla with no specification (176 cases, 17%), and mandible with no specification (87 cases, 8%). Symptoms (n=215) were reported by 72 patients (34%), and local swelling (n=265) was present in 191 cases (72%). Clinical diagnosis included diseases from groups 1, 2 and 3 in, respectively, 863 cases (76%), 244 cases (22%) and 18 cases (2%).

**Figure 1:**
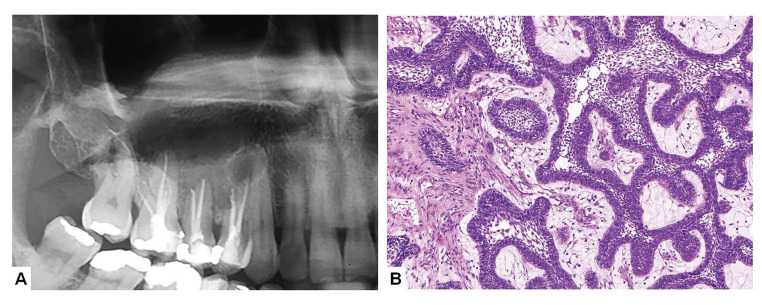
Ameloblastoma resembling an endodontic periapical lesion. (A) Unilocular radiolucent image involving the periapical region of the right maxillary canine, first and second premolars, and first molar; clinical diagnosis was periapical cyst (Note that endodontic treatment was performed in three of these teeth); (B) Histological analysis of the surgical specimen revealed a plexiform ameloblastoma (Hematoxylin and eosin, 400x).

**Figure 2:**
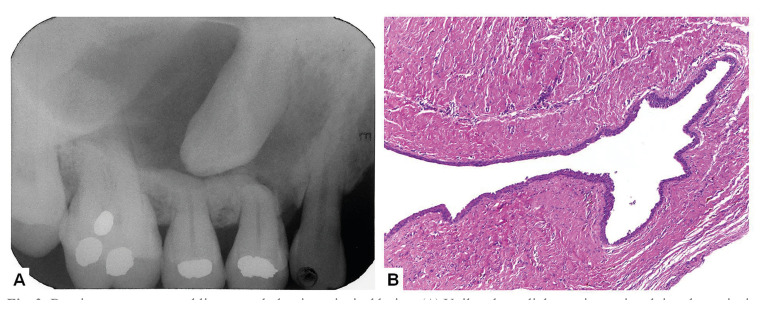
Dentigerous cyst resembling an endodontic periapical lesion. (A) Unilocular radiolucent image involving the periapical region of the right maxillary premolars and in close association with de crown of the unerupted right maxillary canine; clinical diagnosis were periapical cyst and dentigerous cyst; (B) Histological analysis of the surgical specimen revealed a dentigerous cyst (Hematoxylin and eosin, 100x).

Final diagnosis revealed that 917 diagnosis (81%) belonged to group 1, 200 (18%) to group 2 and 8 (1%) to group 3. In group 1, the most common final diagnosis were periapical granulomas and radicular cysts; in group 2, the most common final diagnosis were odontogenic keratocysts, ameloblastomas, nasopalatine cyst, dentigerous cyst and glandular odontogenic cyst (Fig. 1, Fig. 2).

In group 3, final diagnosis included carcinomas (one oral mucosal squamous cell carcinoma infiltrating bone; two poorly differentiated carcinomas arising in the oral mucosa and infiltrating bone; one intraosseous poorly differentiated carcinoma; and one sinusal carcinoma), adenocarcinomas (one metastatic breast carcinoma and one metastatic carcinoma from unknown primary) and melanoma. The diagnosis of non specific odontogenic cyst (70 cases, 33.6%) was rendered to cases in which the clinical and/or radiological information, when provided, together with the histological analysis, were no sufficient to define a specific entity (Table 1).

**Table 1:**
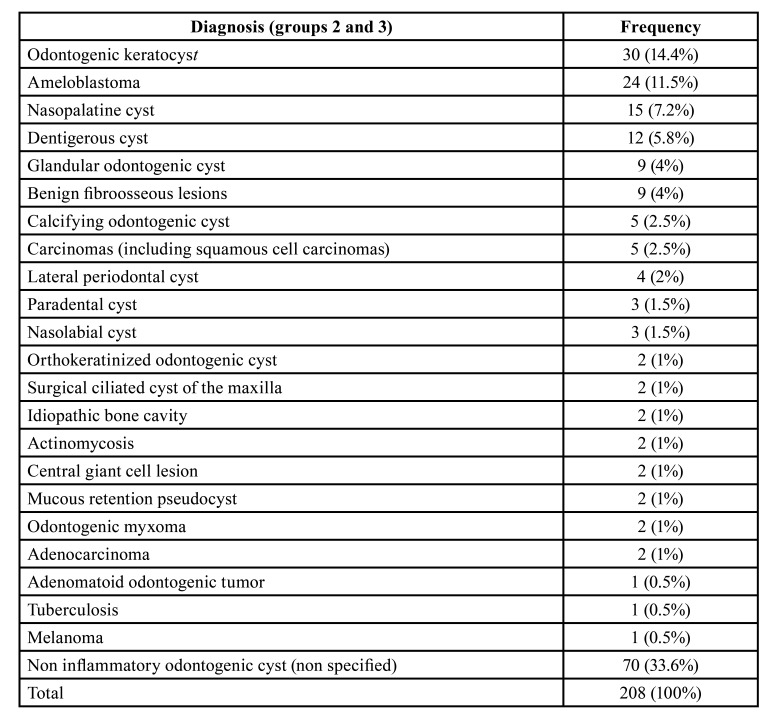
Detailed frequency of the entities diagnosed as nonendodontic periapical lesions.

Mean age of patients in groups 1, 2 and 3 was, respectively, 42.6, 44.8, and 46.9 years (*p*=0.256). Females represented 54% of patients in group 1, males represented 57% of patients in group 2 and there was an equal distribution in group 3 (*p*=0.020). For lesions from group 1, the most common locations were the posterior mandible (28%), anterior maxilla (28%) and posterior maxilla (11%). In group 2, the most common affected regions were also the posterior mandible (33%), anterior maxilla (28%) and posterior maxilla (13%). In group 3, lesions were located in the posterior mandible (50%), anterior mandible (25%) and one case each (12.5%) the posterior and anterior maxilla (*p*=0.001) (Table 2). Symptomatic lesions represented 33%, 32% and 80% of the lesions from, respectively, groups 1, 2 and 3 (*p*=0.082). Local swelling was present in 70%, 76% and 100% of the lesions from, respectively, groups 1, 2 and 3 (*p*=0.681).

The distribution of clinical diagnosis according to the final diagnosis showed that 84% of the lesions classified as group 1 had a clinical diagnosis of a condition also from group 1. In group 2, 53% of the conditions had a clinical diagnosis from the same group; and 75% of the lesions diagnosed in group 3 had a clinical diagnosis of a condition from group 3 (*p*<0.0001) (Table 3).

**Table 2:**
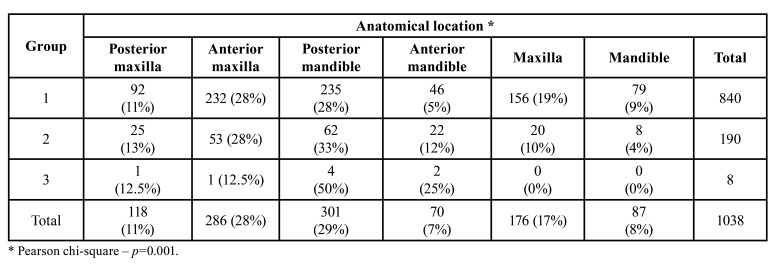
Distribution of the location of the lesions according with the final diagnosis per group (n=1038).

**Table 3:**
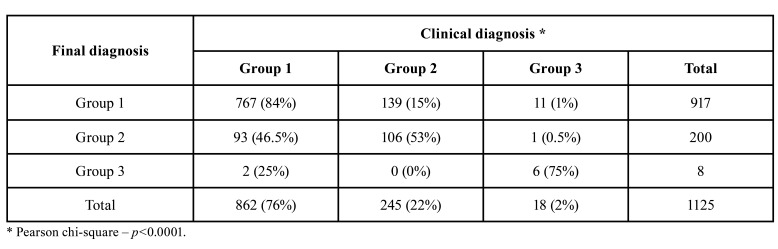
Distribution of the clinical diagnosis according with final diagnosis per group.

## Discussion

EPL are the most common conditions that affect the alveolar bone, but several other NPL can involve the same region, bringing many diagnostic difficulties, especially when located in intimate proximity to the periapical area (5,6). The different methods applied in the case selection for each individual study focusing on NPL turn a direct comparison of the results a hard task. Some studies have included biopsies performed in the periapical region (7-9); others included specimens derived from periapical surgeries indicated to manage supposing EPL refractory to conventional endodontic treatment (10-13); others included biopsies from mandibular and maxillary radiolucent lesions (14); and others included biopsies from the periapical region with a provisional diagnosis of EPL (2,3,5,6), similarly to the present methods.

In all studies, EPL represented the majority of cases, ranging from 73.5% to 99.3% of all cases, including mostly periapical granulomas, radicular cysts, abscesses and fibrous scars. NPL represented, as a consequence, from 0.7% to 26.5% of the samples included in the different studies (Table 4) (2,5-15). In the present study, NPL represented 19% of the periapical lesions that included EPL as at least one of the clinical diagnosis.

**Table 4:**
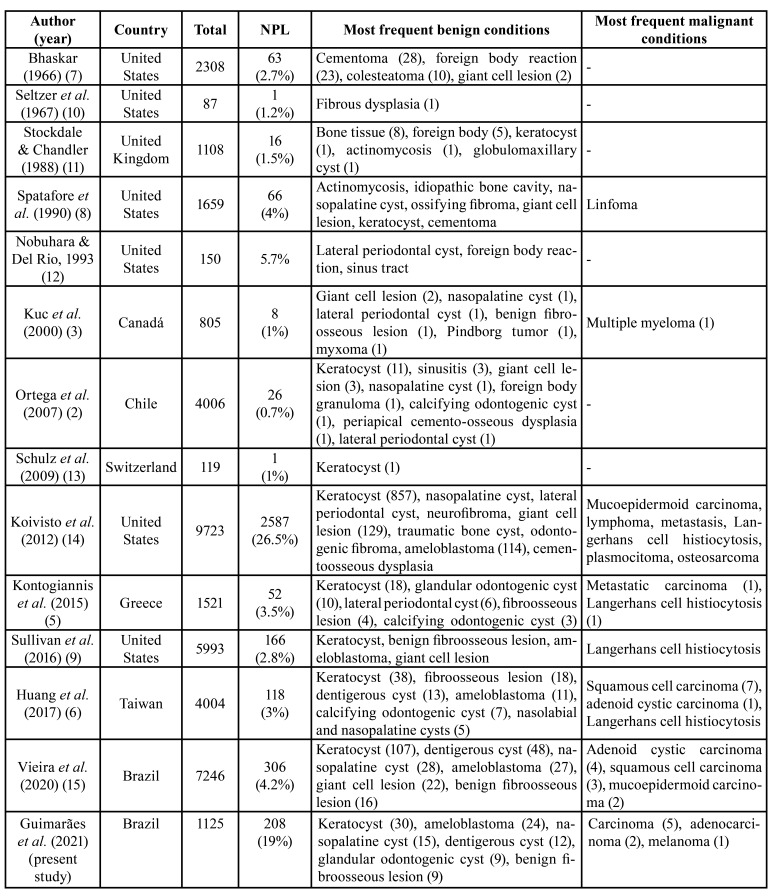
Frequency and distribution of nonendodontic periapical lesions (NPL) reported in the literature.

This high percent is probably associated with the methods used for case selection and seems to mirror the clinical reality of the professionals that submit their biopsies to the respective laboratories, especially clinicians, endodontists, stomatologists, and oral and maxillofacial surgeons. In addition, unfortunately not all EPL are submitted to histological analysis, turning the relative frequency of NPL higher. Anyway, the fact that almost 20% of the cases showed a diagnosis distinct from EPL reinforces the necessity of considering the indication of performing biopsies in cases refractory to conventional endodontic treatment, in periapical lesions that do not have a complete clinical history, and in those presenting uncommon radiological features. It should be also emphasized that all surgical specimens derived from the periapical region should be submitted for histological analysis (2,3,5,9,16). Another fact that reinforces the importance of the NPL is the possibility that teeth situated adjacent to them can coincidentally present with necrotic pulps or previously performed endodontic treatment, turning differential diagnosis with EPL even more difficult (2,4,17).

In general, NPL were more common in patients with a slight higher mean age, affected males more commonly than EPL and showed a higher frequency of involvement of the posterior mandible, as also described in other studies (5,6,14,15). Malignant NPL, in special, were also more associated with the presence of symptoms and local swelling in the affected area (17). The present results highlighted the importance of special attention to the posterior region of the mandible, as malignant NPL showed a predilection for this anatomical region (6).

Most studies have shown that odontogenic keratocysts (32% to 42% of the cases), glandular odontogenic cysts (1% to 19%), benign fibroosseous lesions (4% to 15%), central giant cell lesions (5% to 12%), dentigerous cysts (4 to 11%), lateral periodontal cysts (2% to 12%), ameloblastomas (2% to 9%), calcifying odontogenic cysts (4% to 6%), and nasopalatine cysts (1% to 4%) are the most common NPL (Table 4) (2,5-15).

Odontogenic keratocysts are the most common NPL and represented 14% of the present sample, a lower percentage in comparison to other studies (2,5,6,14,15). One possible explanation to this lower frequency was the inclusion, in the present sample, of cases diagnosed as non inflammatory odontogenic cysts. In these cases, the histological diagnosis confirmation was not possible, as previously explained. The inclusion of these cases is important as it reflects the routine of an Oral Pathology laboratory, calling attention to the difficulties in rendering a final conclusive diagnosis in some cases. This can be due to several factors, such as specimen fragmentation, tissue alterations induced by the inflammatory infiltrate and previous decompression/marsupialization procedures, all of them possibly associated with alterations in the cystic epithelial lining. At least part of the 70 cases diagnosed as non inflammatory cysts included in the present sample could be odontogenic keratocysts. It is also essential to highlight that odontogenic keratocysts show a more agressive and recurrent behavior in comparison to other cysts, and should be included in differential diagnosis of EPL (4,18).

Glandular odontogenic cyst represented 4% of the NPL diagnosed in the present study and should be considered in the differential diagnosis of EPL, especially when dealing with radiolucencies in the anterior mandible (19-21). Kontogiannis *et al*. (5) reported that 19% of the NPL included in their study were glandular odontogenic cysts, but this high frequency has been not reported by any other study.

Benign fibroosseous lesions, mostly the cemento-osseous dysplasias, represent from 4% to 15% of the NPL reported in the literature, particularly their early radiolucent presentation. In the present sample, they represented 4% of the NPL, in contrast to another series (15%) (6). Due to their frequency and the predominant location in the alveolar bone, it is essential to consider these disorders in the differential diagnosis of EPL, especially in Black-skinned adult/older women, avoiding unnecessary endodontic treatment in the adjacent teeth (5,22-25).

Central giant cell lesions were diagnosed in 2 cases in the present sample (1% of the NPL). Dahlkemper *et al*. (26) reinforced that up to 20% of the central giant cell lesions can be associated with the presence of a tooth with pulp necrosis or previous endodontic treatment, being an important differential diagnosis to EPL. Lombardi *et al*. (27) reported 4 central giant cell lesions mimicking EPL, calling attention that previous endodontic treatment had been performed in the associated teeth in 2 of these cases.

Ameloblastomas represented 11.5% of the NPL diagnosed in the present sample, similar to that observed by Huang *et al*. (6). In contrast, ameloblastoma accounted 1.2% of NPL in another study (14). This specific finding can be associated to the fact that both laboratories are references to oral and maxillofacial surgery services. As ameloblastomas should be managed with more extensive surgical procedures, it is essential that these tumors are diagnosed as early as possible (28). Both conventional and unicystic ameloblastomas can mimick EPL, and some reports have shown unnecessary endodontic treatment of the associated teeth, emphasizing the need of considering ameloblastoma in the differential diagnosis of EPL (29-32).

Other odontogenic and nonodontogenic cysts represented a significant part of the present sample, especially the nasopalatine cyst (7.2%), dentigerous cyst (5.8%), calcifying odontogenic cyst (2.5%), and lateral periodontal cyst (2%). These findings are close to the ones reported by other studies (2,5). Conversely, Huang *et al*. (6) reported that 11% of NPL were dentigerous cysts. Nasopalatine cysts are classically located in the maxillary midline and can be superimposed to the roots of the central incisors mimicking EPL (33,34). Dentigerous cysts, when associated to mixed dentition or when located laterally to the tooth crown, can be superimposed to the roots of adjacent erupted teeth, simulating EPL (35). Calcifying odontogenic cysts with early calcifications can be characterized by exclusive radiolucent images and should be also included in the differential diagnosis of EPL (36). Lateral periodontal cysts are predominantly located between the roots of two erupted teeth and should be always considered in the differential diagnosis of lateral EPL (37,38).

Malignant neoplasms represented 3.6% to 7.6% of the NPL included in different studies (Table 4) (6,15). Pontes *et al*. (17) revised 56 cases of NPL (from their own files and from the literature) and showed that 29% were malignant tumors. This frequency is, however, superstimated as the authors revised mostly case reports in which the possibility of including rare cases is higher. Most studies have shown that squamous cell carcinomas (2.6% to 6%), adenocarcinomas (1%), metastasis, lymphomas, mucoepidermoid carcinomas, and Langerhans cell histiocytosis, are the most common malignant NPL (5,14). In contrast, Vieira *et al*. [15] reported that adenoid cystic carcinomas were the most common malignancies in their study. It is important to highlight that oral and maxillofacial metastasis, mostly from primary tumors from lungs, breast, kidney and prostate, have a predilection for the posterior mandible and gingiva and could mimick EPL (39). Additionally, due to the presence of pain and secondary infection, gingival squamous cell carcinomas could also simulate periapical abscesses (40).

One interesting point observed in the present series and not discussed in most previous studies is the comparison of clinical provisional and final histological diagnosis. The present results showed that 16% of the cases initially interpreted as EPL, 47% of the cases initially interpreted as benign NPL, and 25% of the cases initially diagnosed as malignant NPL, received the final diagnosis of a condition from another group. In accordance with these findings, Kontogiannis *et al*. (5) reported that in 50 out of their 52 NPL, the clinical differential diagnosis did not include the final diagnosis. This reinforces the necessity of adequate anamnesis and oral examination, and the correct indication of clinical, radiological and surgical complementary exams, when establishing the correct differential diagnosis of EPL versus NPL.

The frequency and distribution of NPL show heterogeneous results when comparing distinct populations. These differences can be attribuTable to specific characteristics from each population, to the profile of the professionals who send the specimens to histological analysis, to the experience of the pathologist, and to the specific methods used for data retrieval and interpretation in each individual study. In this way, understanding the profile from each population and comparing data from different studies are essential to apply the provided information to each specific population, focusing on early identification and establishment of proper management to the NPL.

In conclusion, the results from the present study showed that NPL were identified in 19% of the periapical lesions included in the present sample. Benign NPL were mostly odontogenic keratocyst, ameloblastomas, nasopalatine cyst, dentigerous cyst, glandular odontogenic cysts and benign fibroosseous lesions. Malignant NPL included carcinomas, adenocarcinomas and melanoma. Accurate clinical and imaging examination are essential to consider a NPL in the differential diagnosis of periapical radiolucencies. Periapical lesions with unusual imaging features or that do not heal after root canal treatment should be submitted to incisional biopsy or excised, and sent for histopathological analysis.
